# Designing a new biosensor “DNA ELISA” to detect *Escherichia coli* using genomic DNA and comparison of this method to PCR-ELISA

**DOI:** 10.1080/14756366.2018.1450748

**Published:** 2018-04-04

**Authors:** Elaheh Hashemi, Mehdi Forouzandeh

**Affiliations:** Department of Medical Biotechnology, Tarbiat Modares University, Tehran, Iran

**Keywords:** DNA-ELISA, probe, *Escherichia coli*, hybridisation

## Abstract

In this experiment, DNA-ELISA biosensor was introduced, bearing the ability to detect specific bacteria in about 4 h. This is a more rapid system in comparison to conventional methods, like colony counting method. Moreover, this method does not require any amplification and directly detects genomic DNA of bacteria, giving a lower limit to the sensitivity of 40,000 bacteria. In this study, two specific probes capture (biotin labelled) and detector (dig labelled), were used against special regions of 16s rRNA gene of *Escherichia coli* ATCC 25922. The capture probe has the ability to trap the target bacterial DNA from a pool of other kinds of bacteria under specific conditions. The detector probe then was used to hybridize to the genome of trapped bacteria. The detection proceeds by adding HRP-anti dig enzyme and its substrate, ABTS to emit light. Light absorbance is measured for verifying the detection.

## Introduction

**Figure 1.**
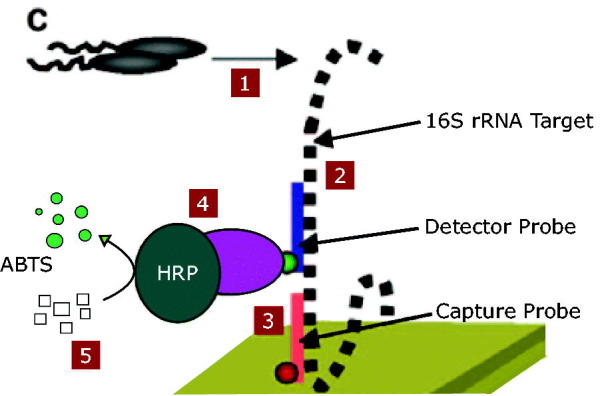
Illustration of the DNA-ELISA method. 1. The bacteria were cultured in LB. 2. The detector probe hybridized to 16 s rRNA gene of the target bacteria. 3. Capture probe conjugate to the complementary sequence in the16s rRNA gene. 4. HRP enzyme added to the solution. 5. ABTS resulted in colour for detection (This figure is adopted by Joseph C. Lia^4^).

Bacterial pathogens are distributed in soil, marine and intestinal tract of humans. Humans carry more than 150 kinds of bacteria^1^. Among these bacteria, some can have profound effect on humans and animals and may cause different infectious disease. Infectious disease account for nearly 40% of total 50 million annual estimated deaths^1–3^. Pathogen detection is the utmost significance for health and safety reasons. Among these pathogen bacteria, Enterobacteriaceae, especially *Escherichia coli,* involved in infectious diseases such as urinary tract infections (UITs). *Escherichia coli* is the most common and thoroughly studied model bacterium^4–7^. The effective detection of bacteria needs method of analysis that meets a lot of challenging criteria. Time and sensitivity of analysis are the most important limitation, associated to the value of microbiological testing^1^^,^^2^. Traditional approaches for analysis of *E. coli* have relied on cultural techniques and many selective-differential media that have been developed. These methods usually were based on a morphological evaluation of the microorganism and require growth of single cell in to a colony. This process is time consuming because making a colony containing 10^6^ organism will take between 18 and 24 h even in some cases up to 72 h to obtain confirmed results^1^^,^^5–7^. The steps of traditional methods involve, pre-enrichment, selective enrichment, biochemical screening and serological confirmation^1^. Molecular diagnostic experiments like PCR-based methods need fewer number of primary cell but they call for equipment and expert technical personnel. Besides, amplification of cells in PCR needs lot of time before screening^7–9^. For this reason, diagnosis of a pathogen always requires a rapid and low cost method. The DNA hybridisation technology has opened new trends for improving detection methods with more selectivity and less time and money consuming^9–11^. The goal of this study was to design a rapid, selective and sensitive method for pathogen detection. For this purpose, hybridisation of specific probe to the special complementary region of *E. coli* gene was used in place of amplification. Detection occur directly from genome. Our approach is outlined in Figure 1. In brief, after hybridisation of dig labelled probe to its complementary region of 16 s rRNA of *E. coli* ATCC 25922, the substrates’ colour of HRP-anti dig (ABTS) turn to green so the presence of bacteria can be detected directly with faster speed.

## Materials and methods

### Bacterial strains

Bacterial species *E. coli* ATCC 25922 used for the test. For cross reaction these bacteria were used: *Klebsiella pneumonia* ATCC 70603, *Staphylococcus haemolyticus* PTCC 1437, *Streptococcus pyogenes* PTCC 1447, *Enterococcus faecalis* ATCC 29212, *Pseudomonas aeruginosa* ATCC 27863, *Proteus mirabilis* ATCC 25933, *Serratia marcescens* ATCC 13880, *E. coli* PTCC1270, *E. coli* PTCC 1276. All the bacteria were obtained from Iranian Research Organization for Science and Technology (IROST). The bacteria were subcultured in Tryptic Soy Broth (TSB) and bacterial stocks were prepared by 70% glycerol. Bacteria were subcultured in Luria broth (LB) at 37 °C overnight, until it gets the OD (600) of 1 that is approximately 5 × 10^8^ to 1 × 10^9^ cells/ml^4^.

### DNA extraction

About 1–2 ml cultured bacteria were transferred in to 2-ml tube and the DNA of the bacteria extracted based on INTRON biotechnology company kit protocol (Korea). DNA concentration was adjusted to 240 ng/µl.

### Probe and primer design

Two specific probes (capture and detector) for the special region of 16 s rRNA in *E. coli* ATCC 25,922 were selected based on the sequence reported by Joseph C. Lia^4^. The capture probe was biotin labelled at 5′´ end to enable conjugation with the streptavidin coated in test plate (5′-GTCAATGAGCAAAGGTATTAACTTTACTCCCTTCC-3′). Detector probe contains dig at 5′ end that can attach to antidig-HRP enzyme (5′ CTGAAAGTACTTTACAACCCGAAGGCCTTCTTCAT-3′). Two 20 oligonucleotides primer (16 sf and 16 sr) were design by means of pick primer tools in NCBI for sequencing of the gene. Primers were generated for the region contains probe sequence and their performance, including self-complementary and GC percent, were evaluated by oligo program. “16 sf” Primer was located between bp 124–143. “16 sr” primer was located between bp 880–899 at the 16 s rRNA gene. The probes were located between bp 399–433 (detector) and bp 440–474 (capture). Another dig labelled 309 bp detector probe was designed by using the former 35mer detector probe and 16sf primer in PCR.

### Producing PCR product for PCR-ELISA test

PCR was performed for 35 cycles. Samples were incubating at 95 °C for 15 s to denature the DNA, then at 58 °C for 30 s to annealed the primers and finally 72 °C for 60 s to extend the annealed primers. Labelling of the PCR-generated fragment was performed with Digoxigenin-11-2′-deoxy-uridine-5′-triphosphate, alkali- stable kit (USA Roche company) by adding dig in PCR samples according to the manufacture’s instruction.

### Producing PCR product for developing detector probe

35-nucleotide detector probe did not result enough OD for detection so a it needs probe with more dig on it. For this purpose, PCR product was produced by using the aforementioned procedure and using the 35-nucleotide detector probe as reverse primer. The sample contains 16sf primer as forward primer, 35-nucleotide detector probe, template DNA and master mix.

### PCR-ELISA test

The procedure of PCR-ELISA has been done by using the PCR-ELISA dig detection (Roche company) protocol. PCR product was incubated for 10 min in 15–20 °C in denaturation buffer contain NAOH. About 250 µl of mixture containing Capture probe and hybridisation buffer, was added to the former solution. Then the solution was added to the streptavidin coated wells and incubated for 3 h at 37 °C with shaking. After aspirating and washing the wells three to five times with washing buffer, 200 µl of HRP-anti dig enzyme was added to the wells and incubate for 30 min in 37 °C with shaking. Again wells aspirated and washed three to five times with washing buffer and 200 µl of ABTS solution was added. The absorbance of the solutions was measured in 405 nm after 30 min incubation in darkness.

### DNA-ELISA test

The procedure of DNA-ELISA has been done by using the PCR-ELISA dig detection (Roche company) protocol. DNA and 309 bp detector probe were incubated for 10 min in 15–20 °C in denaturation buffer contain NAOH. About 250 µl of mixture containing Capture probe and hybridisation buffer was added to the former solution. About 200 µl of the solution including two probe, DNA and buffers was added to the streptavidin coated wells and incubated for 3 h at 37 °C with shaking. After aspirating and washing the wells three to five times with washing buffer, 200 µl of HRP-anti dig enzyme was added to the wells and incubate for 30 min in 37 °C with shaking. Again wells aspirated and washed three to five times with washing buffer and 200 µl of ABTS solution was added. The absorbance of the solutions was measured in 405 nm after 30 min incubation in darkness (Figure 1).

## Results

### DNA-ELISA

#### Preparation of 300 bp, dig labelled detector probe

A 35-nucleotide detector probe with just one dig at the end was used as primer. The presence of prepared 300 bp, dig labelled probe, was proved by the appearance the band on gel agarose. The labelled PCR product was a little upper than the unlabelled.

#### Optimizing the capture and detector probes

To optimize the concentration of probes in the experiment, different dilution of probe as shown in Tables 1 and 2 for DNA-ELISA, were tested. The most reasonable which can create more colour in comparison to the concentration of probe, was detected for the experiment (Tables 1 and 2). For capture probe the concentration of 20 pm/µl was selected because as the concentration increases, the OD differences become lower. For detector probe the last concentration was selected because using more detector probe could unbalance the other concentration of solution. Also in this concentration we can get enough OD for detection.

**Table 1.** Optimization capture probe.

Sample number	DNA (µl)	Capture probe (µl)	Detector probe (µl)	OD
1	5	0.25	5	0.16
2	5	0.5	5	0.20
3	5	1	5	0.22
4	5	2	5	0.23
Negative control	5	–	5	0.075

Concentration of capture probe: 10 pm/µl, concentration of detector probe: 400 ng/µl, concentration of DNA: 240 ng/µl.

**Table 2.** Optimization detector probe.

Sample number	DNA (µl)	Capture probe (µl)	Detector probe (µl)	OD
1	5	2	2.5	0.18
2	5	2	5	0.28
3	5	2	10	0.36
4	5	2	20	0.50
Negative control	5	2	–	0.08

Concentration of capture probe: 10 pm/µl, concentration of detector probe: 400 ng/µl, concentration of DNA: 240 ng/µl.

#### Sensitivity

To determine the sensitivity of the tests serially diluted extracted DNA were prepared. Tenfold serial dilution of a known concentration (240 ng/µl) were used directly for DNA-ELISA test and as template for PCR-ELISA test. The detection limit of the *E. coli* detection was 0.024 ng/µl that is around 10^4^ bacteria for DNA-ELISA (Table 3). The absorbance in this concentration was 0.14 that was upper than cut off value^11^ for the test (0.11).

**Table 3.** Sensitivity of the test.

Sample number	DNA (ng)	Capture probe (µl)	Detector probe (µl)	OD
1	240	2	20	0.40
2	24	2	20	0.31
3	2.4	2	20	0.26
4	0.24	2	20	0.14
5	0.024	2	20	0.01
Negative control	–	2	20	0.08

Serial dilution of DNA was tested. Concentration of capture probe: 10 pm/µl, concentration of detector probe: 400 ng/µl, initial concentration of DNA: 240 ng/µl.

#### Evaluation of test with other bacteria and determine the specificity

To verify the specificity of the tests nine other bacteria that is more common in UTIs was subjected to the experiment. Equal concentration of extracted DNA of nine bacteria including two *E. coli* tested (*K. pneumonia, S. haemolyticus, S. pyogenes, E. faecalis, P. aeruginosa, P. mirabilis, S. marcescens, E. coli* PTCC1270, *E. coli* PTCC 1276). For the DNA-ELISA test, the extracted DNA was used directly. As shown in Table 4 the two *E. coli* were positive and has visible absorbance with green colour but other bacteria are negative and the colourless.

**Table 4.** Evaluation of specificity.

Organisms	DNA (µl)	Capture probe (µl)	Detector probe (µl)	OD
*Klebsiella pneumonia*	10	2	10	0.04
*Staphylococcus haemolyticus*	˝	˝	˝	0.03
*Streptococcus pyogenes*	˝	˝	˝	0.04
*Enterococcus faecalis*	˝	˝	˝	0.09
*Pseudomonas aeruginosa*	˝	˝	˝	0.08
*Proteus mirabilis*	˝	˝	˝	0.08
*Serratia marcescens*	˝	˝	˝	0.03
*E. coli* PTCC1270	˝	˝	˝	0.20
*E. coli* PTCC 1276	˝	˝	˝	0.24
Negative control	˝	˝	˝	0.04

Nine common bacteria in UTI including 2 *E. coli* with PTCC number were subjected to the test. Absorbencies are shown in OD column.

#### Assessment of repeatability of the test by inter and intra assays

The repeatability of the test was evaluated by inter- and intra-assays (Table 5). Triplicates of special concentrations in different times of a day for intra assay were tested by DNA-ELISA. The means of acquired OD for three days used for inter assay. The standard deviation and coefficient of variation of the test were calculated. Intra-assay coefficient of variations was 3.84–9.80% and intra-assay coefficient of variations were 5.26–7.14%, respectively.

**Table 5.** Evaluation of repeatability of the DNA-ELISA test.

Inter assay		
Days	Mean ± SD (240 ng DNA)	%CV
1	015/. ± 396/.	3/84
2	.023/. ± 407/	5/65
3	020/. ± 406/.	4/92
3 × 3	021/. ± 399/.	5/26
Intra assay		
Number of repetition in one day	Mean ± SD (240 ng DNA)	%CV
3	015/. ± 396/.	84/3

Inter- and intra-assays.

### PCR-ELISA

#### Optimizing the capture probes

To optimize the concentration of probe in PCR-ELISA different dilution of probe as shown in Table 6, were tested and the most reasonable one that can create more colour in comparison to the concentration of probe was detected for the experiment. For capture probe the last concentration was selected because using more detector probe could unbalance the other concentration of solution. Also in this concentration we can get enough OD^1^ for detection.

**Table 6.** Optimization capture probe for PCR-ELISA.

Sample number	PCR product (µl)	Capture probe (µl)	OD
1	5	0.25	0.5
2	5	0.5	0.6
3	5	1	0.8
4	5	2	1
Negative control	5	–	0.08

Concentration of capture probe: 10 pm/µl, concentration of template DNA for PCR: 240 ng/µl.

#### Sensitivity

To determine the sensitivity of the PCR-ELISA serially diluted extracted DNA were used as template for producing PCR product. Tenfold serial dilution of a known concentration (240 ng/µl) was used for the test. The detection limit for PCR-ELISA was 0.0024 ng/µl that it is around 10^3^ bacteria for the test (Table 7). The absorbance in this concentration was 0.5 that was upper than Cut off value^11^ for the test (0.11).

**Table 7.** Sensitivity of the PCR-ELISA.

Sample number	DNA (ng)	Capture probe (µl)	OD
1	240	2	1
2	24	2	0.8
3	2.4	2	0.6
4	0.24	2	0.26
5	0.024	2	0.2
Negative control	–	2	0.08

Serial dilutions of DNA were used as template for the tested. Concentration of capture probe: 10 pm/µl. Initial concentration of DNA: 240 ng/µl.

#### Evaluation of test with other bacteria and determine the specificity

**Figure 2.**
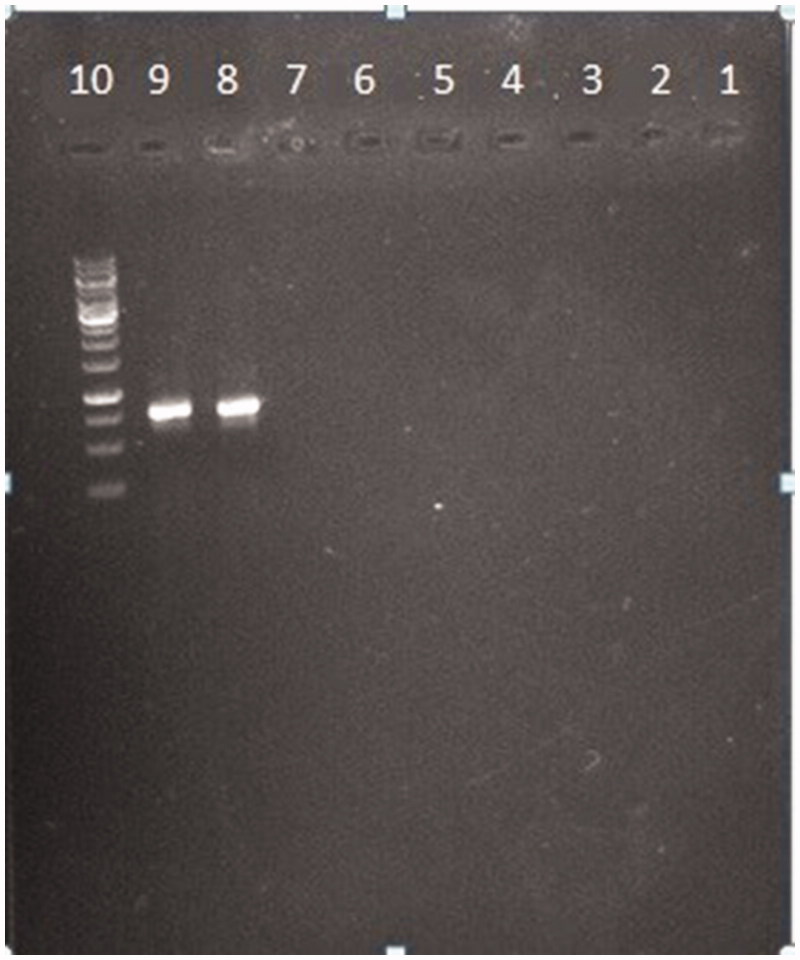
Evaluation of specificity. Nine common bacteria in UTI including 2 E. coli with PTCC number were subjected to the PCR. Each number is related to each of the bacteria specimen. (1)* Klebsiella pneumonia. ATCC 70603*. (2)* Staphylococcus haemolyticus PTCC 1437*. (3)* Streptococcus pyogenes PTCC 1447*. (4)* Enterococcus faecalis ATCC 29212*. (5)* Pseudomonas aeruginosa ATCC 27863*. (6)* Proteus mirabilis ATCC 25933*. (7)* Serratia marcescens ATCC 13880*. (8)* E. coli PTCC1270*. (9)* E. coli PTCC 127*.

To verify the specificity of the tests nine other bacteria that is more common in UTIs were subjected to the experiment. Equal concentration of extracted DNA of nine bacteria including two *E. coli* were used as template for preparing PCR-product (*K. pneumonia, S. haemolyticus, S. pyogenes, E. faecalis, P. aeruginosa, P. mirabilis, S. marcescens, E. coli* PTCC1270, *E. coli* PTCC 1276). The results show that just the DNA of two *E. coli* have ability to appear a band on gel so, they will have absorbance in PCR-ELISA test (Figure 2).

## Discussion

A number of other studies have been carried out to detect pathogens^1^^,^^2^^,^^5^^,^^12^. Detection of *E. coli* is important because, in most of UTIs cases, *E. coli* causes the disease^4^. In all the traditional and new methods exist for detection of this bacterium, molecular-based methods are become widespread due to the sensitivity and specificity. Among the molecular methods, the ones which are based on hybridisation of probes, have been using more and more^13–16^. Also using probe obtain reasonable specificity. Safe materials like fluorescent (real time PCR) and enzymes (PCR-ELISA) can be used instead of ethidium bromide in gel^17–21^. Methods like growing on culture and using microscope for detection are so long and takes at least 24 h. Besides, visual detection on microscope is not reliable. PCR-ELISA and real time also need expensive equipment and experts.

In current study, we introduced a novel “DNA-ELISA” method, bearing the ability to detect specific bacteria in about 4 h. The method offers simplicity, ease of use, minimal hand on time and does not need any special equipment and experts. Moreover, DNA-ELISA does not require any amplification and can directly detect genomic DNA of bacteria, giving a lower limit to the sensitivity of 40,000 bacteria. Although the detection limit for DNA-ELISA is much more than PCR-ELISA, it reduces the longevity of the detection by elimination the amplification step significantly. At present there is an ongoing effort to improve the sensitivity of detection limit.

## References

[1] Ivnitski D, Abdel-Hamid I, Atanasov P, Wilkins E. Biosensors for detection of pathogenic bacteria. Biosens Bioelectron 1999;14:599–624. 10.1016/s0956-5663(99)00004-4 10230031

[2] Leonard P, Hearty S, Brennan J, et al Advances in biosensors for detection of pathogens in food and water. Enzyme Microb Technol 2003;32:3–13.

[3] Chen S, Cheng YF. Biosensors for bacterial detection. Int J Biosens Bioelectron 2017;2:00048.

[4] Liao JC, Mastali M, Gau V, et al Use of electrochemical DNA biosensors for rapid molecular identification of uropathogens in clinical urine specimens. J Clin Microbiol 2006;44:561–70. 16455913 10.1128/JCM.44.2.561-570.2006 PMC1392664

[5] Lazcka O, Del Campo FJ, Munoz FX. Pathogen detection: a perspective of traditional methods and biosensors. Biosens Bioelectron 2007;22:1205–17. 16934970 10.1016/j.bios.2006.06.036

[6] Jothikumar N, Griffiths MW Rapid detection of *Escherichia coli* O157:H7 with multiplex real-time PCR assays. Appl Environ Microbiol 2002;68:3169–71. 12039787 10.1128/AEM.68.6.3169-3171.2002 PMC123919

[7] Lee S, Yoo S. Optical biosensors for the detection of pathogenic microorganisms. Trends Biotechnol 2016;34:7–25. 26506111 10.1016/j.tibtech.2015.09.012

[8] Rola M, Kuzmak J The detection of bovine leukemia virus proviral DNA by PCR-ELISA. J Virol Methods 2002;99:33–40. 11684301 10.1016/s0166-0934(01)00384-6

[9] Bakthavathsalam P, Rajendran VK, Mohammed JAB. A direct detection of *Escherichia coli* genomic DNA using gold nanoprobes. J Nanobiotechnol 2012;10:1–10. 10.1186/1477-3155-10-8 PMC3306828 22309695

[10] Shingo U, Hirotoshi E, Po-Sung C, et al Detection of bacterial DNA by in situ hybridization in patients with decompensated liver cirrhosis. BMC Gastroenterol 2017;17:106. 29041907 10.1186/s12876-017-0664-z PMC5646152

[11] Salazar N, Caetano-Anollés G. Nucleic acid scanning-by-hybridization of enterohemorrhagic Escherichia coli isolates using oligodeoxynucleotide arrays. Nucl Acids Res 1996;24:5056–7. 9016682 10.1093/nar/24.24.5056 PMC146331

[12] Nayak M, Kotian A, Marathe S, Chakravortty D. Detection of microorganisms using biosensors—a smarter way towards detection techniques. Biosens Bioelectron 2009;25:661–7. 19782558 10.1016/j.bios.2009.08.037

[13] Lamture JB, LBeattie K, Burke BE, et al Direct detection of nucleic acid hybridization on the surface of a charge coupled device. Nucl Acids Res 1994;22:2121–5. 8029021 10.1093/nar/22.11.2121 PMC308130

[14] Rohde A, Hammerl JA, Appel B, et al FISHing for bacteria in food-A promising tool for the reliable detection of pathogenic bacteria?. Food Microbiol 2015;46:395–407. 25475309 10.1016/j.fm.2014.09.002

[15] Rohde A, Hammerl JA, Appel B, et al Differential detection of pathogenic *Yersinia* spp. by fluorescence in situ hybridization. Food Microbiol 2017;62:39–45. 27889163 10.1016/j.fm.2016.09.013

[16] Fortin NY, Mulchandani A, Chen W Use of real-time polymerase chain reaction and molecular beacons for the detection of *Escherichia coli* O157:H7. Anal Biochem 2001;289:281–8. 11161323 10.1006/abio.2000.4935

[17] Kawasaki S, Fratamico PM, Kamisaki-Horikoshi N, et al Development of the multiplex PCR detection kit for *Salmonella* spp., listeria monocytogenes, and *Escherichia coli* O157: H7. JARQ 2011;45:77–81.

[18] Laitinen R, Malinen E, Palva A. PCR-ELISA: I: application to simultaneous analysis of mixed bacterial samples composed of intestinal species. Syst Appl Microbiol 2002;25:241–8. 12353879 10.1078/0723-2020-00118

[19] Malinen E, Mättö J, Salmitie M, et al PCR-ELISA: II: analysis of *Bifidobacterium* populations in human faecal samples from a consumption trial with *Bifidobacterium lactis* Bb-12 and a galacto-oligosaccharide preparation. Syst Appl Microbiol 2002;25:249–58. 12353880 10.1078/0723-2020-00117

[20] de Boer E, Beumer RR. Methodology for detection and typing of foodborne microorganisms. Int J Food Microbiol 1999;50:119–30. 10488848 10.1016/s0168-1605(99)00081-1

[21] Singh G. Determination of cutoff score for a diagnostic test. Internet J Lab Med 2007;2:1–4.

